# Tumor necrosis factor, interleukin-1 and interleukin-8 mediate the nociceptive activity of the supernatant of LPS-stimulated macrophages

**DOI:** 10.1080/09629359791686

**Published:** 1997-06

**Authors:** S. M. Thomazzi, R. A. Ribeiro, D. I. Campos, F. Q. Cunha, S. H. Ferreira

**Affiliations:** 1 Department of Physiology and Pharmacology Center of Health Sciences Federal University of Ceará Fortaleza CE 60430-270 Brazil; 2 Department of Pharmacology Faculty of Medicine of Ribeirão Preto University of São Paulo Ribeirão Preto SP 14049-900 Brazil

## Abstract

It has been suggested that the supernatant of LPSstimulated macrophages (macrophage nociceptive factor, MNF) promotes nociception in mice. Intraperitoneal administration of MNF induced dose-related writhing, which reached a plateau between 18 and 26 min after injection and decreased within 60 min. The release of MNF was inhibited by the pretreatment of the macrophages with cycloheximide, a protein synthesis inhibitor, or with the glucocorticoid dexamethasone. Cyclooxygenase inhibitors, such as indomethacin or paracetamol, had no effect. The MNF-induced nociception was inhibited in a dose-related manner by pretreatment of the animals with indomethacin, paracetamol or dexamethasone. Pretreatment of the animals with the sympatholytics guanethidine and atenolol partially reduced the MNF nociception, which was abolished by the combination of guanethidine or atenolol with indomethacin. The preincubation of MNF with antisera against TNF-α, IL-1 or IL-8 partially inhibited its nociceptive effect. Intraperitoneal injection of a mixture of the recombinants cytokines TNF-α, IL-1 and IL-8 mimicked MNF nociception. The individual injection of these cytokines was unable to induce the nociceptive effect. In conclusion, our data suggest that the nociceptive activity of the supernatant of LPSstimulated macrophages is explained by the presence of TNF-α, IL-1 and IL-8, the nociceptive activity of which (in mice) seems to be due to the release of cyclooxygenase and sympathetic metabolites.


Mediators of Inflammation, **6**, 195–200 (1997)

### Tumor necrosis factor, interleukin-1 and interleukin-8 mediate the nociceptive activity of the supernatant of LPS-stimulated macrophages

S. M. Thomazzi,[1] R. A. Ribeiro,[1] D. I. Campos,[2] F. Q. Cunha[2,CA] and S. H. Ferreira[2]

[1]Department of Physiology and Pharmacology, Center of Health Sciences, Federal University of Ceará, 60430-270 Fortaleza, CE; and [2]Department of Pharmacology, Faculty of Medicine of Ribeirão Preto, University of São Paulo, 14049-900 Ribeirão Preto, SP, Brazil

[CA]Corresponding Author
Fax: (+55) 16 633 2301
Email: fdqcunha@fmrp.usp.br

**It has been suggested that the supernatant of LPS-stimulated macrophages (macrophage nociceptive factor, MNF) promotes nociception in mice. Intraperitoneal administration of MNF induced dose-related writhing, which reached a plateau between 18 and 26 min after injection and decreased within 60 min. The release of MNF was inhibited by the pretreatment of the macrophages with cycloheximide, a protein synthesis inhibitor, or with the glucocorticoid dexamethasone. Cyclooxygenase inhibitors, such as indomethacin or paracetamol, had no effect. The MNF-induced nociception was inhibited in a dose-related manner by pretreatment of the animals with indomethacin, paracetamol or dexamethasone. Pretreatment of the animals with the sympatholytics guanethidine and atenolol partially reduced the MNF nociception, which was abolished by the combination of guanethidine or atenolol with indomethacin. The preincubation of MNF with antisera against TNF-α, IL-1 or IL-8 partially inhibited its nociceptive effect. Intraperitoneal injection of a mixture of the recombinants cytokines TNF-α, IL-1 and IL-8 mimicked MNF nociception. The individual injection of these cytokines was unable to induce the nociceptive effect. In conclusion, our data suggest that the nociceptive activity of the supernatant of LPS-stimulated macrophages is explained by the presence of TNF-α, IL-1 and IL-8, the nociceptive activity of which (in mice) seems to be due to the release of cyclooxygenase and sympathetic metabolites.**

**Key words:** Cytokines, Macrophages, Nociception

### Introduction

It has been proposed that two biochemical pathways are involved in inflammatory pain which are dependent on the release of cyclooxygenase (mainly $PGE_2$ and $PGI_2$) or sympathetic metabolites (mainly dopamine), respectively. It is assumed that the contribution of each pathway depends on the characteristics of the nociceptive stimulus. These two components were demonstrated in rats using a paw pressure test,[1] in mice using the writhing test,[2] and in guinea pigs by nociception evoked by electrical stimulation.[3]

Cytokines seem to constitute a link between cellular injury and recognition of non-self and the development of local and systemic inflammatory signs and symptoms (e.g. cell migration, oedema, fever and hyperalgesia[4–8]). In this context, we have recently demonstrated that in carrageenan-evoked hyperalgesia, a cascade of cytokine release preceded the generation of cyclooxygenase products and sympathomimetic mediators.[5,9,10] It was suggested that the initial cytokine released is TNF-α which induces either: IL-1β and IL-6 release, which in turn stimulate the production of cyclooxygenase products; or IL-8 release which stimulates production of sympathomimetic mediators.[10]

We have previously shown that macrophage monolayers stimulated with endotoxin (LPS) release a factor with an apparent MW greater than 10 kDa into the culture medium which, when injected intraperitoneally into mice, induced writhing. This macrophage nociceptive factor was referred to as MNF; induction of writhing by MNF was inhibited by pretreatment of mice with indomethacin; dipyrone, but not indomethacin, blocked its release by LPS-stimulated macrophage monolayers.[11]

In the present study we investigated the effect of the cyclooxygenase inhibitor paracetamol and the glucocorticoid dexamethasone on

© 1997 Rapid Science Publishers 

MNF release by LPS-stimulated macrophage monolayers.[11] The effect of indomethacin and paracetamol, as well as sympatholytic agents, such as atenolol and guanethidine, on MNF-induced nociception was also determined to investigate the involvement of cytokines in MNF activity; we tested the effect of antibodies against TNF-α, IL-1β or IL-8 on MNF nociceptive activity.

### Materials and Methods

### Animals

Male Wistar rats, 150–180 g body weight, were used to obtain the peritoneal macrophages, and male Swiss mice, 20–25 g bodyweight, were used for the nociceptive test. The animals were housed in a temperature controlled room ($23 \pm 2$°C) with free access to water and food. The ethical guidelines as described in the NIH Guide for the Care and Use of Laboratory Animals was followed throughout the experiments described.

### MNF production

Peritoneal macrophages were obtained from Wistar rats 4 days after intraperitoneal (i.p.) injection of 10 ml thioglycollate (3% w/v). The cells were harvested with 10 ml of RPMI medium and allowed to adhere to plastic tissue culture dishes for 1 h at 37°C ($2 \pm 0{\cdot}2 \times 10^6$), in a $CO_2$ incubator as previously described.[11] After 1 h of incubation, non-adherent cells were removed by washing three times with phosphate buffered saline (PBS) pH 7.4. The adherent population, consisting of $95 \pm 2$% macrophages (determined by conventional light microscopy after staining with Rosenfeld pancromic stain), was then incubated for 30 min at 37°C in fresh medium (control) or medium containing 5 µg/ml of *Escherichia coli* lipopolysaccharide (LPS). The supernatant was discarded and the monolayers were washed three times with PBS and then incubated for 1 h in 4 ml of medium without LPS. At the end of incubation the viability of the macrophages was determined by trypan blue exclusion (viability higher than 90%) and the cell-free incubation fluids were ultrafiltered through an Amicon YM-10 membrane (which restricts substances of a molecular weight greater than 10 kDa). The residue retained by the membrane was washed with PBS and dilutions were made in order to obtain a solution in which 1 ml originated from $4 \times 10^6$ macrophages. On the basis of its biological action, the material obtained (as described above) is referred to as macrophage nociceptive factor (MNF).

### Nociceptive test

The nociceptive activity of MNF was tested in mice using the writhing model.[12] For this purpose, 0·1 ml/g body weight of MNF solution was injected into the peritoneal cavity of the test animals. The intensity of nociception was quantified by counting the total number of writhes occurring between 14 and 34 min after MNF injection. In the experiment, in which the time course of MNF activity was determined, the number of writhes was counted between 10 and 50 min.

### Effect of different drugs on MNF release and on its nociceptive activity

The effects of indomethacin ($10^{-5}$ M and $10^{-4}$ M) paracetamol ($10^{-4}$ M) dexamethasone ($10^{-5}$ M) and cyclohexamide ($10^{-5}$ M and $3 \times 10^{-5}$ M) on MNF release *in vitro* were tested by adding these drugs to the incubation fluid 30 min before LPS challenge of the macrophage monolayers. During the LPS stimulation periods as well as during the incubation period, the drugs were also present. The possible effect of these drugs present in the supernatant of the writhing response could be disregarded, since the supernatants were ultrafiltered on an Amicon YM-10 membrane before being injected into the animals.

The effects of indomethacin (0.5 and 2·0 mg/kg, 30 min before), paracetamol (100 and 200 mg/kg, 30 min before), atenolol (0·5 mg/kg, 30 min before), and dexamethasone (0.5 and 2·0 mg/kg, 30 min before) on MNF-induced nociception were tested, treating the animals by subcutaneous (s.c.) injection. To investigate the effect of guanethidine (30 mg/kg) on MNF activity, the animals were pretreated daily by the s.c. route for 3 days before MNF injection.

### Effects of anti-cytokine sera on MNF activity

The antisera (anti-human IL-1β, anti-murine TNF-α, anti-human IL-8) and the control serum were incubated with MNF solution for 10 min before injection of the mixture into the test mice. In an earlier experiment, we demonstrated that these anti-sera neutralized the hyperalgesic effects of their respective rat cytokines.[10]

### Nociceptive effect of TNF-α, IL-1β and IL-8

TNF-α (0·5–100 ng/mouse), IL-1β (0·005–100 ng/mouse) and IL-8 (0·1–50 ng/mouse) were injected i.p. into the test mice and the number of writhes was determined between 14 and 34 min afterwards. The effects of the combination of these cytokines (100 ng of IL-1 + 100 ng of TNF-α + 50 ng of IL-8 per mouse), and their dilutions (1:3 and 1:9) were also tested.

### Drugs, reagents and antisera

The following drugs were used: atenolol (Sigma, USA; Atn); bradykinin (Sigma, USA; BK); cycloheximide (Sigma, USA; CHX); dexamethasone (Merck, Sharp and Dohme, USA; Dexa); endotoxin (*E. coli* lipopolysaccaride; Difco, USA; LPS); guanethidine (Sigma, USA; Gua); indomethacin (Merck, Sharp and Dohme, ISA; Indo); paracetamol (Sigma, USA; Ptol); thioglycolate (Difco, USA). The medium used was RPMI 1640 (Difco, USA). TNF-α, IL-1β and IL-8 (72 amino acids) were NIBSC preparations coded 87/650, 86/680 and 89/520, respectively. The following antisera were used: sheep anti-human IL-1β (Poole *et al.*, 1989[13]), sheep anti-human IL-8 (Cunha *et al.*, 1991[9]) and sheep anti-murine TNF-α (Mahadevan *et al.*, 1990[14]); these were kindly provided by Dr S. Poole (NIBSC, England, UK).

### Statistical analysis

Analysis of variance (ANOVA) followed by Bonferroni's test were used. Statistical differences were considered to be significant at $P < 0{\cdot}05$.

### Results

### Nociceptive effect of LPS-stimulated macrophage supernatant (MNF)

The i.p. injection of supernatant from rat macrophage monolayers incubated at 37°C with LPS (5 μg/ml) into mice induced a dose-related nociception, which reached the plateau between 18 and 26 min after injection (Fig. 1A). Supernatants obtained from non-stimulated macrophages or from LPS-stimulated macrophages incubated at 4°C, did not induce the nociceptive response (Fig. 1B). Similar results were obtained with the supernatant of the mice macrophages stimulated with LPS (data not shown).

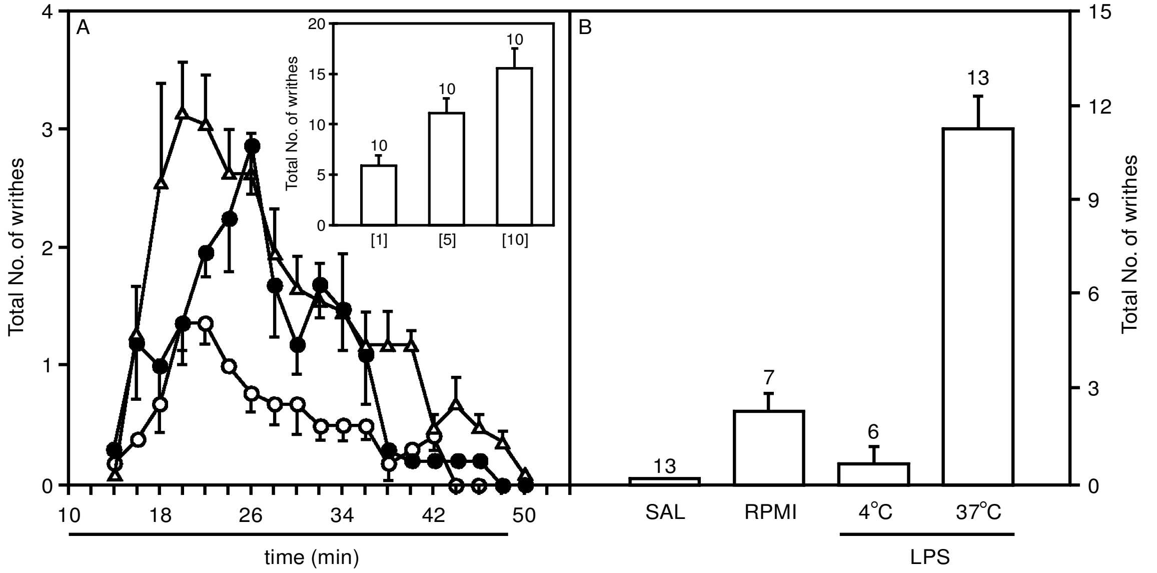

FIG. 1. Nociceptive effect of the supernatant of LPS-stimulated macrophages (MNF, macrophage nociceptive factor). (A) Time course (10–50 min) of the nociceptive response induced by different doses of MNF: △, 1 ml/g body weight; ●, 0·1 ml/g; ○, 0·01 ml/g). The insert in (A) shows the total number of writhes determined in the interval between 14 and 34 min after injection of MNF at doses of 0·01 ml/g body weight [1], 0·1 ml/g [5] and 1 ml/g [10]. (B) Number of writhes induced by i.p. injeciton of: SAL, saline; RPMI, supernatant of macrophages incubated with medium alone; LPS 4°C/LPS 37°C, supernatant of macrophages stimulated for 30 min with LPS (5 μg/ml) and incubated at 4°C or at 37°C (0·1 ml/g body weight). The number of writhes was determined in the interval between 14 and 34 min after MNF injection. In all panels the number at the top of each bar represents the number of mice in that experimental group. The results are reported as means ± SEM. *, Statistically significant differences (P < 0·05) between the group and the control group (SAL) determined by ANOVA and Bonferroni's test.

### Effects of cycloheximide, dexamethasone, indomethacin and paracetamol on MNF release

The treatment of macrophage monolayers with the protein synthesis inhibitor cycloheximide ($10^{-5}$, $3 \times 10^{-5}$ M) or with the glucocorticoid dexamethasone ($10^{-5}$ M), but not with the cyclooxygenase inhibitors, indomethacin ($10^{-5}$–$10^{-4}$ M) or paracetamol ($10^{-4}$ M), diminished MNF activity in LPS-stimulated macrophage supernatants (Fig. 2).

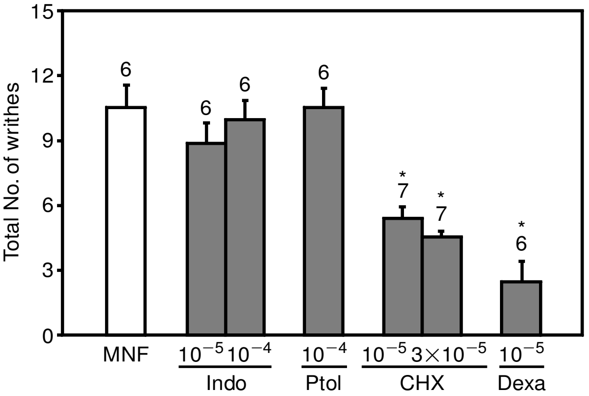

FIG. 2. Effects of indomethacin, paracetamol, cycloheximide and dexamethasone on MNF release. The bars show the number of writhes induced by the supernatant (0·1 ml/g body weight, i.p.) from LPS-pretreated macrophages in the absence (MNF) or the presence of the indicated concentration (M) of indomethacin (Indo), paracetamol (Ptol), cycloheximide (CHX) or dexamethasone (Dexa). The number at the top of each bar represents the number of mice in that group. The values are reported as means ± SEM of the total number of writhes occurring between 14 and 34 min after MNF injection. *, Statistically significant differences ($P < 0.05$) from the control group determined by ANOVA and Bonferroni's test).

### Effects of cyclooxygenase inhibitors, sympathetic blockers and dexamethasone on MNF activity

The subcutaneous administration of indomethacin (0.5 and 2·0 mg/kg), paracetamol (100 and 200 mg/kg) or dexamethasone (0.5 and 2·0 mg/kg) inhibited the nociception induced by MNF (Fig. 3A). Fig. 3B shows that although the sympathetic blockers guanethidine (Gua, 30 mg/kg, daily) and atenolol (Atn, 0·5 mg/kg) partially inhibited the nociceptive effect of MNF, the effects were not significant. However, the combination of guanethidine or atenolol with a dose of indomethacin (0·5 mg/kg), that partially inhibited MNF activity, abolished the nociceptive effect of MNF (Fig. 3C). The administration of the higher doses of the drugs used (indomethacin, paracetamol, guanethidine, atenolol or dexamethasone) in normal animals did not promote any behavioural alteration.

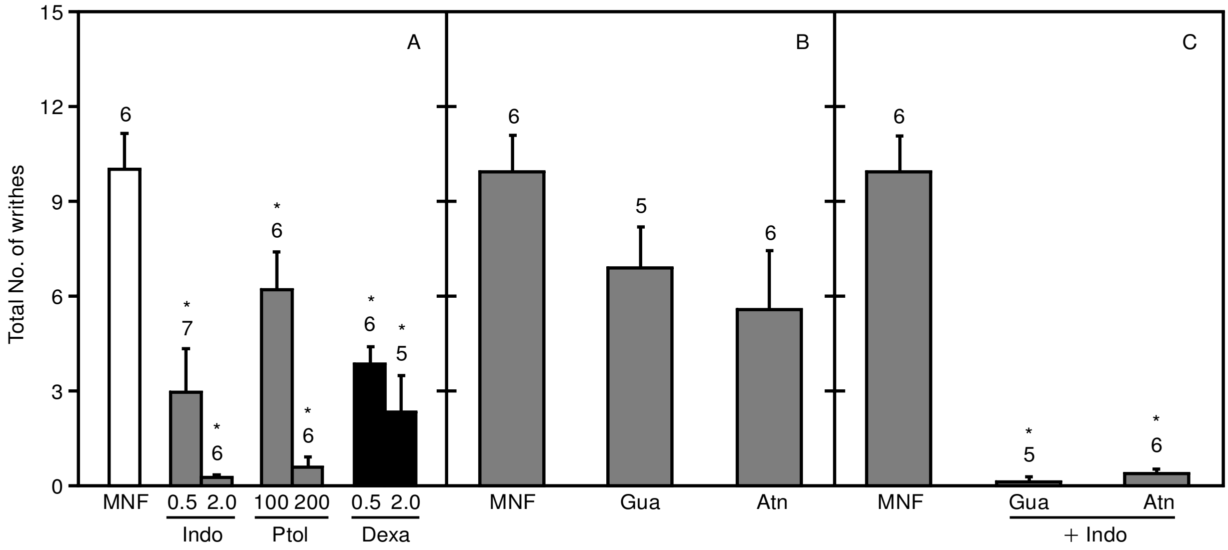

FIG. 3. Effects of indomethacin, paracetamol, dexamethasone, guanethidine and atenolol on MNF-induced writhing in mice. (A) Number of writhes induced by MNF in untreated mice (MNF, i.p., 0·1 ml/g body weight) and in mice pretreated s.c. with the indicated doses (mg/body weight) of indomethacin (Indo, 30 min before), paracetamol (Ptol, 30 min before) or dexamethasone (Dexa, 60 min before) (B) Number of writhes induced by MNF in untreated mice (MNF, i.p., 0·1 ml/g body weight) and in mice pretreated by s.c. injection of guanethidine (Gua, 30 mg/kg) or atenolol (Atn, 0·5 mg/kg) 30 min before injection of the nociceptive stimulus (MNF). (C) Number of writhes induced by MNF in untreated mice (MNF, i.p., 0·1 ml/g body weight) and in mice pretreated by s.c. injection of the association of guanethidine (Gua, 30 mg/kg) and indomethacin (Indo, 0·5 mg/kg) and atenolol (Atn, 0·5 mg/kg) 30 min before the injection of the nociceptive stimulus (MNF). The values are reported as means ± SEM of the total number of writhes occurring between 14 and 34 min after challenge with MNF. The number at the top of each bar represents the number of mice in that group. *, Statistically significant differences ($P < 0.05$) from the control group determined by ANOVA and Bonferroni's test.

### Inhibitory effects of anti-cytokine sera on the nociceptive activity of MNF

Preincubation of MNF with antisera (50 μl) against IL-8, IL-1β or TNF-α significantly inhibited its nociceptive activity. Control serum did not affect MNF activity (Fig. 4). Antiserum against IL-6 did not affect MNF-activity.

### Nociceptive effects of IL-1β, IL-8 and TNF-α

Intraperitoneal injection of different doses of IL-1 (0·005–100 ng/mouse), IL-8 (0·1–50 ng/mouse) or TNF-α (0·5–100 ng/mouse) did not induce significant nociceptive responses in mice. However, like MNF, the combination of these cytokines (100 ng of TNF-α and IL-1 plus 50 ng IL-8) induced a significant nociceptive response. This combination, when diluted 3- or 9-fold, induced only small nociceptive response (Fig. 5).

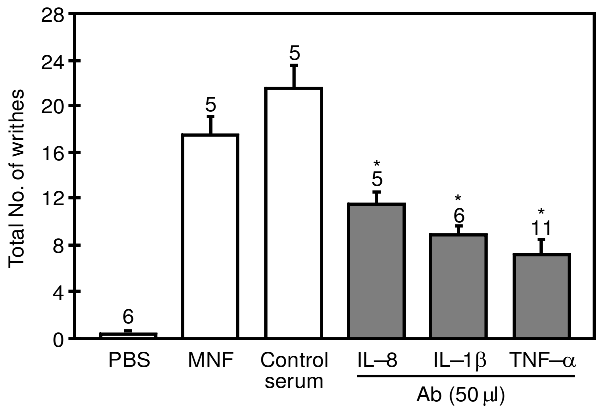

FIG. 4. Effect of antisera against TNF-α, IL-1β or IL-8 on the nociception induced by MNF. The MNF samples were incubated with 50 μl of the anti-cytokine sera or with control serum for 10 min and then injected i.p. The values are reported as means ± SEM of the total number of writhes occurring between 14 and 34 min after challenge with MNF alone or in combination with the sera. PBS, number of writhes induced by PBS. The number at the top of each bar represents the number of mice in that group. *, Statistically significant differences (P < 0·05) from the positive control group (MNF) determined by ANOVA and Bonferroni's test.

### Discussion

We have confirmed our previous results showing that macrophage monolayers stimulated with LPS (5 μg/ml) at 37°C release a nociceptive factor (MNF) into the supernatant that induces dose-dependent writhing in mice.[12] The absence of the nociceptive activity in the super-

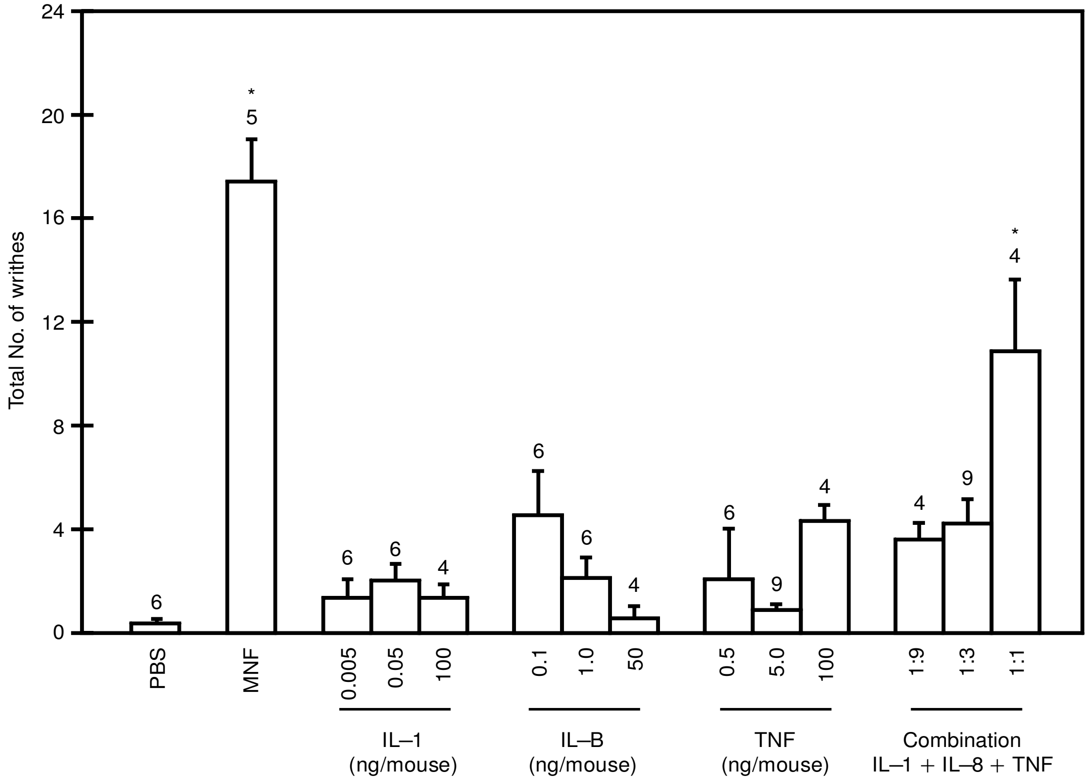

FIG. 5. The nociceptive effects of IL-1β, IL-8, TNF-α or their mixture. The mice were injected with PBS, MNF, and indicated doses (ng/mouse) of IL-1, IL-8, and TNF-α or with three doses of the mixture of these three cytokines. The highest dose of the mixture was 100 ng/mouse IL-1β + 50 ng/mouse IL-8 + 100 ng/mouse TNF-α (1:1); this was then used to prepare 1:3 or 1:9 dilutions. The number of writhes was determined in the interval between 14 and 34 min after the stimulus injection. The number at the top of each bar represents the number of mice in that group. The values are reported as means ± SEM. *, Statistically significant differences (P < 0·05) from the negative control (PBS) determined by ANOVA and Bonferroni's test.

natant when the LPS-stimulated macrophages were incubated at 4°C eliminates the possibility that the observed effect is due to LPS contamination. The nociceptive activity of MNF reached a plateau between 18 and 26 min, and decreased thereafter.

It was also confirmed that MNF is not a cyclooxygeanse product,[11] since the cyclooxygenase inhibitors, indomethacin or paracetamol, did not affect its release. Furthermore, the presence of arachidonic acid metabolites in the MNF preparation could be disregarded since the ultrafiltration step used in the preparation of MNF excludes substances with a MW smaller than 10 kDa. The fact that the protein synthesis inhibitor cycloheximide and the glucocorticoid dexamethasone blocked the release of MNF by LPS-stimulated macrophages suggests that it is a protein. Since glucocorticoids are classical inhibitors of cytokine activity, and LPS-stimulated macrophages release various cytokines, including IL-1, IL-8 and TNF-α, which promote hyperalgesia,[9,10] we investigated the possibility that the MNF activity is due to the presence of these cytokines in the MNF samples. Using an effective concentration of antiserum against IL-1, IL-8 or TNF-α,[9,10] we observed that all the sera tested partially inhibited the nociceptive activity of MNF, suggesting that MNF activity may be due to the combined nociceptive activities of IL-1, IL-8 and TNF-α present in the MNF samples. The presence of these cytokines in the supernatants of LPS-stimulated macrophages used to prepare MNF has been described.[15,16] The possibility that MNF activity results from the combination of these three cytokines was tested by injection of different concentrations of these cytokines alone or in combination into the abdominal cavity of mice. Although IL-1, IL-8 and TNF-α injected singly at high doses were unable to promote a significant nociception in mice, injection in combination reproduced the MNF-induced nociception.

The nociception caused by MNF was blocked in a dose-dependent manner by the cyclooxygenase inhibitors indomethacin and paracetamol, as well as by dexamethasone. The sympathetic blockers guanethidine and atenolol also partially reduced the effect of MNF, and their combination with a low dose of indomethacin abolished MNF nociception. These results indicate that nociception induced by MNF is the result of the local release of sympathetic and cyclooxygenase mediators. The data show, however, that the presence of cyclooxygenase metabolites are essential triggering MNF activity, since indomethacin and paracetamol abolished MNF nociception. The inhibition by dexamethasone of MNF activity can be explained by its ability to block the release of prostaglandin via inhibition of phospholipase $A_2$,[17,18] as well as the expression of cyclooxygenase 2.[19]

In conclusion, we demonstrated that the nociceptive activity of the supernatant of LPS-stimulated macrophages may be due to the presence of IL-1, IL-8 and TNF-α. These cytokines induce nociception mainly by the activation of the prostaglandin component of inflammatory pain, although the activation of sympathetic components may be also involved.

### References

1. Nakamura M, Ferreira SH. A peripheral sympathetic component in inflammatory hyperalgesia. *Eur J Pharmcol* 1987; **135**: 145–153.
2. Duarte IDG, Nakamura M, Ferreira SH. Participation of the sympathetic system in acetic acid-induced writhing in mice. *Braz J Med Biol Res* 1988; **231**: 341–342.
3. Nakamura M, Lico MC. Peripheral analgesic effect of clonidine in awake guinea pigs. *Braz J Med Biol Res* 1985; **17**: 406–409.
4. Dinarello CA, Cannon JG, Wolff, *et al.* Tumor necrosis factor (cachectin) is an endogenous pyrogen and induces production of interleukin 1. *J Exp Med* 1986; **163**: 1443–1449.
5. Ferreira SH, Lorenzetti BB, Bristow AF, Poole S. Interleukin-1β as a potent hyperalgesic agent antagonized by a tripeptide analogue. *Nature* 1988; **334** (6184): 698–699.
6. Faccioli LH, Souza GEP, Cunha FQ, Poole S, Ferreira SH. Recombinant interleukin-1 and tumor necrosis factor induce neutrophil *in vivo* by indirect mechanisms. *Agents Actions* 1990; **30** (3/4): 344–349.
7. Ribeiro RA, Flores CA, Cunha FQ, Ferreira SH. IL-8 causes *in vivo* neutrophil migration by a cell-dependent mechanism. *Immunology* 1991; **73**: 472–477.
8. Dinarello CA. The proinflammatory cytokines interleukin-1 tumour necrosis factor and treatment of septic shock syndrome. *J Infect Dis* 1991; **163**: 1177–1184.
9. Cunha FQ, Lorenzetti BB, Poole S, Ferreira SH. Interleukin-8 as a mediator of sympathetic pain. *Br J Pharmacol* 1991; **104**: 765–767.
10. Cunha FQ, Poole S, Lorenzetti BB, Ferreira SH. The pivotal role of tumour necrosis factor α in the development of inflammatory hyperalgesia. *Br J Pharmacol* 1992; **107**: 660–664.
11. Campos DI, Cunha FQ, Ferreira SH. A new mechanism of action of dipyrone: blockade of the release of a nociceptive factor from macrophages. *Braz J Med Biol Res* 1988; **21**: 565–568.
12. Collier HOJ, Dinneen LC, Johnson CA, Schneider C. The abdominal constriction response and its suppression by analgesic drugs in the mouse. *Br J Pharmacol Chemother* 1968; **32**: 295–310.
13. Poole S, Bristow AF, Selkirk S, Rafferty AE. Development and application of radioimmune assays for Interleukin-1α and Interleukin-1β. *J Immunol Methods* 1989; 116: 259–264.
14. Mahadevan V, Malik STA, Meager A, Fiers W, Lewis GP, Hart IR. Role of tumour necrosis factor in flavone acetic-induced tumour vasculature shutdown. *Cancer Res* 1990; 50: 5537–5542.
15. Ribeiro RA, De Lucca FL, Flores CA, Cunha FQ, Ferreira SH. RNA from LPS-stimulated macrophages induces the release of tumour necrosis factor-α and interleukin-1 by resident macrophages. *Mediators of Inflammation* 1993; **2**: 435–442.
16. Ribeiro RA, Flores CA, Cunha FQ, Ferreira SH, De Lucca FL. Partial characterisation of the RNA from LPS-stimulated macrophages that induces the release of chemotactic cytokines by resident macrophages. *Mol Cell Biochem* 1995; **148**: 105–113.
17. Blackwell GJ, Carnuccio R, Di-Rosa M, Flower RJ, Parente L, Perisco P. Macrocortin: a polypeptide causing the anti-phospholipase effect of glucocorticoid drugs. *Nature* 1980; **297**: 147–149.
18. Flower RJ. Lipocortin and the mechanism of action of glucocorticoids. *Br J Pharmacol* 1988; **94**: 987–1015.
19. Masferrer JL, Zweifel BS, Manning PT, *et al.* Selective inhibition of inducible cyclooxygenase 2 *in vivo* is anti-inflammatory and nonulcerogenic. *Proc Natl Acad Sci* 1994; **91**: 3228–3232.

ACKNOWLEDGEMENTS. We are indebted to Neomesia Issajuara Freiri, and Fabiola Mestriner for technical assistance. This work was supported by grants from FAPESP and CNPq (Brazil).

**Received 19 February 1997; accepted in revised form 25 March 1997.**
